# Validation of the Chinese version of the low physical activity questionnaire (LoPAQ) with ActiGraph accelerometer in hemodialysis patients

**DOI:** 10.1186/s12882-021-02230-3

**Published:** 2021-01-08

**Authors:** Huang Rui, Zhang Haifen, Yang Yan, Fang Nina, Liu Qian, Ma Jun, Wang Min, Shi Ling, Tao Xingjuan

**Affiliations:** 1 Shanghai Jiao Tong University School of Nursing, No.227, South Chongqing Rd, Shanghai, 200025 China; 2 Department of Biomedical Engineering, National University of Singapore, Singapore, Singapore; 3 Nursing Department, Renji Hospital, School of Medicine, Shanghai Jiao Tong University, Shanghai, China; 4 Department of Nephrology, Tong Ren Hospital, Shanghai Jiao Tong University School of Medicine, Shanghai, China

**Keywords:** Hemodialysis, Physical activity, Validity, Reliability, Accelerometer

## Abstract

**Background:**

Valid instruments for measuring physical activity at the low end of the physical activity range and producing quantitative results are required among dialysis patients who are extremely inactive. This study aimed to translate and adapt a Chinese version of the low physical activity questionnaire (LoPAQ) and to examine its reliability and validity among hemodialysis patients.

**Methods:**

This was a cross-sectional study. The LoPAQ was translated into Chinese and culturally adapted following the standardized questionnaire adaptation process. Participants wore an ActiGraph for seven consecutive days and were asked to complete the Chinese version of the LoPAQ (C-LoPAQ) following the ActiGraph monitoring period. The criterion validity of the C-LoPAQ was examined with accelerometers using Spearman’s correlation coefficients. Bland-Altman plots were adopted to determine the absolute agreement between methods. The test-retest reliability was analyzed using the intraclass correlation coefficient (ICC).

**Results:**

Eighty-five hemodialysis patients had valid accelerometers and C-LoPAQ data. The total walking time reported on LoPAQ was correlated with step counts by ActiGraph (*rho* = 0.47, *p* < 0.01). A moderate correlation was also observed between the C-LoPAQ and the ActiGraph-measured physical activity for total calories (*rho* = 0.44, *p* < 0.01). There was a fair correlation between ActiGraph-measured sedentary time and C-LoPAQ-measured inactive time (*rho* = 0.22, *p* < 0.05). The test-retest reliability coefficients of C-LoPAQ ranged from 0.30 to 0.66.

**Conclusions:**

The C-LoPAQ demonstrated moderate validity for measuring low levels of physical activity, especially walking, and total kilocalories of physical activity among hemodialysis patients in China.

**Supplementary Information:**

The online version contains supplementary material available at 10.1186/s12882-021-02230-3.

## Background

Chronic kidney disease (CKD) has become a public health issue, affecting approximately 8–16% of the adult population worldwide [1]. The global prevalence was estimated to be 9.1% in 2017. In China, the number of CKD patients reached 132.3 million in 2017 [2]. With irreversible disease progression, numerous CKD patients will ultimately progress to end-stage renal disease (ESRD), which necessitates renal replacement therapy including maintenance hemodialysis (MHD) and peritoneal dialysis (PD) [3].

In MHD patients, a low physical activity level is closely related to poor prognosis [4]. Several studies have confirmed the relationship between a low physical activity level and the increased risk of cardiovascular disease in MHD patients [5, 6], which was the leading cause of morbidity and mortality in these patients [7, 8]. Beddhu et al. [9] reported a 40% higher mortality rate in CKD patients with low physical activity levels than in those with higher levels. Accordingly, appropriate moderate physical activity helps prevent muscle loss, control comorbidities, improve quality of life, and reduce the mortality rate in MHD patients [10, 11].

Physical activity refers to all skeletal muscle movements that result in energy consumption. It can be classified into low, moderate, and high levels according to energy consumption [12, 13]. Patients treated with MHD generally have decreased physical activity levels [14]. A study by Johansen et al. [15] showed that the physical activity level of MHD patients was significantly lower than that of the healthy sedentary population, especially in the elderly population, where the discrepancy reached 57% in those in their 70s. Additionally, research has shown that the physical activity level of MHD patients would progressively decline within the whole course of the disease on a scale of approximately 4.5% per month [16]. In addition, Li et al. [17] found that traffic- or housework-related activities composed the largest part of the energy consumption of MHD patients, which meant that the activity types of MHD patients were usually limited to low classification [18].

The common questionnaires for evaluating physical activity include the 7-day Physical Activity Recall Questionnaire (PAR), Human Activity Profile (HAP), and International Physical Activity Questionnaire (IPAQ), which have been used in CKD patients in previous studies [15, 16, 18]. However, these questionnaires are designed for general adults, either focusing on moderate-to-high-level activities or lacking necessary accuracy for quantized and dynamic clinical evaluation [19, 20]. Prior literature has documented that physical activity even at very low levels is strongly associated with survival among dialysis patients [21].

The low physical activity questionnaire (LoPAQ) developed by Johansen et al. [22] emphasizes walking behavior (representing low-level physical activity), quantifies the calorie consumption of all leisure activities, and takes sitting time as one of the negative evaluation criteria. These designs make LoPAQ a good fit for MHD patients, considering the physical activity characteristics of MHD patients mentioned above. Furthermore, the questionnaire enables researchers to estimate whether an MHD patient has reached the activity level recommended by the guidelines on a rather precise scale. Moreover, the English version of the LoPAQ has good validity and is highly correlated with the Minnesota Leisure Time Activity Questionnaire and various indicators of physical function [22]. Currently, the LoPAQ is only available in English. To the best of our knowledge, there is still no Chinese version of the LoPAQ being applied in MHD patients. The purpose of this study was to translate and adapt the LoPAQ in Chinese and determine its reliability and validity in MHD patients.

## Methods

### Study design and participants

This was a cross-sectional study. Patients undergoing hemodialysis treatment were recruited from outpatient hemodialysis units of two hospitals (two hemodialysis units in Renji Hospital and one hemodialysis unit in Tongren Hospital, both associated with the Shanghai Jiao Tong University School of Medicine) in mainland China through convenience sampling from December 2018 to March 2019. The inclusion criteria were as follows: 1) age ≥18 years; 2) on hemodialysis for ≥3 months; 3) able to walk without assistance; and 4) ability to provide informed consent and complete the questionnaires. The exclusion criteria were as follows: 1) diagnosis of mental or cognitive disorders; 2) unstable conditions; and 3) hospitalization in the previous 3 months. The study was approved by the Human Subjects Ethical Sub-committee of the Shanghai Jiao Tong University (SJUPN-201705). Written informed consent was obtained from all participants prior to data collection.

### Low physical activity questionnaire (LoPAQ)

The original English version of the LoPAQ was developed by Johansen et al. (Supplementary File 1) [22], focusing on physical activities at a low level for HD patients. The questionnaire comprises 11 items assessing the parameters of physical activity within the last 7 days, which include minutes of walking around the neighborhood, for fitness or pleasure and for transportation, as well as the average time spent on sedentary and sitting activities. The questionnaire calculates kilocalories expended in light, moderate, vigorous, and total physical activities. The validity of the English version of the LoPAQ was supported by its substantial correlations with the Minnesota Leisure Time Activity Questionnaire (*rho* = 0.62, *p* < 0.001), the Physical Function score of the 36-item Short Form Health Survey (SF-36; *rho* = 0.64, *p* < 0.001), and physical performance indexes [22].

### Translation and cultural adaption of the Chinese version of the LoPAQ (C-LoPAQ)

Cross-cultural adaptation was performed according to the steps recommended by the World Health Organization [23]. After obtaining author approval for linguistic adaption and validation, the original English version of the LoPAQ was forward-translated to Chinese by two independent bilingual experts who were fluent in English but whose native language was Chinese. An expert panel including three nursing researchers who were fluent in English agreed to a compatible version of C-LoPAQ after comparing two translated versions and addressing the unambiguity of each item. The C-LoPAQ was blindly back-translated to English independently by two other bilingual translators who earned doctoral degrees (one with a doctoral degree in linguistics and another with a doctoral degree in nursing) in English-speaking countries. The translators had no prior knowledge of the original LoPAQ. The back-translated English questionnaire was compared to the original English version by the expert panel. Any discrepancies and inconsistencies between the two versions were adjusted and conformed with the original author until all ambiguities disappeared. An expert committee, including three senior clinical renal physicians and two nurses, was formed to determine the idiomatic and conceptual equivalence of the C-LoPAQ.

### Measurements

#### ActiGraph

Physical activity was assessed using an ActiGraph GT3X+ (ActiGraph, Pensacola, FL). It is a small, watch-like, unobstrusive device that measures acceleration in three axes. The device was initialized using 60-s epochs to collect data at a sampling rate of 30 Hz. Participants were instructed to wear the ActiGraph on the right hip on an elastic waistband during waking hours, except when bathing, swimming, or doing other water activities, for seven consecutive days. On the last data collection day, a research assistant went to the dialysis unit to remove the accelerometer. Raw accelerometer data were downloaded using ActiLife 6 software (ActiGraph) and were analyzed in 60-s epochs. The default filter settings were adopted. Wear time was validated using a filter of 150 consecutive zero-count minutes, with allowance for < 1 min of activity counts < 100 counts per minute [24]. At least one dialysis day and two non-dialysis days with a minimum of 8 h of wear time were adopted as the criteria for valid ActiGraph data [25]. The triaxial vector magnitude counts per minute cut-off for different physical activity intensities were determined as follows: sedentary behavior, < 99; light, 100–1951; moderate, 1952–5724; and vigorous, > 5725.

#### Demographic and clinical information

A questionnaire on the demographic and clinical information of the patients, consisting of two parts, was self-developed. Part I included demographic information such as age, sex, education, marital status, residence, and living conditions. Part II recorded the clinical data, including the cause of chronic renal failure and dialysis vintage.

### Procedures

After obtaining written consent to participate in the study, each participant completed a demographic questionnaire during the dialysis session and received an ActiGraph for 7 days. After 7 days, the research assistant visited the dialysis center again to retrieve the ActiGraph. On the ActiGraph removal day, the research assistant administered the C-LoPAQ face-to-face. The participants were asked to recall their physical activity and sedentary behavior over the past 7 days for the same time the ActiGraph was worn. All clinical information was extracted from the inpatient information system of the hospital. After two weeks, the participants were invited to complete the C-LoPAQ again to evaluate test-retest reliability.

### Data analysis

Statistical analysis was performed using SPSS 21.0 software (IBM, Armonk, NY). Demographic information was presented using descriptive statistics, including mean, standard deviation (SD), and percentage. The levels of skewness and kurtosis were determined to assess the normality of each variable [26]. Content validity of the questionnaire was measured using the content validity index (CVI) and content validity ratio (CVR). The item-level CVI (I-CVI) was calculated as the percentage of specialists giving a rating of either 3 or 4 [27]. The scale-level CVI (S-CVI) was computed as the items on the questionnaire that obtained a rating of 3 or 4, divided by the total item numbers [28]. Generally, a CVI higher than 0.78 and a CVR higher than 0.75 suggest good content validity [29]. Spearman’s correlation coefficients were used to examine the relationships between subscales of the C-LoPAQ and ActiGraph parameters. A correlation of above 0.40 is considered acceptable [30]. Bland-Altman analyses were used to determine the level of agreement for total energy expenditure per week, derived from the C-LoPAQ and ActiGraph. The internal consistency of the C-LoPAQ was assessed using Cronbach’s α. The test-retest reliability was determined by calculating the intraclass correlation coefficient (ICC, two-way mixed analysis of variance). An ICC > 0.75 represents an “excellent” test-retest reliability, 0.60–0.74 represents “good,” 0.40–0.59 represents “fair,” and < 0.4 indicates “poor” test-retest reliability [31]. All statistical tests were two-tailed, and *p* < 0.05 was considered statistically significant.

## Results

### Characteristics of study population

A total of 96 participants were recruited for the validity test. A total of 85 MHD patients who had valid ActiGraph and C-LoPAQ data were included in the data analysis, among whom 56 (65.9%) were from Renji Hospital (58.9 and 41.1% for each hemodialysis unit) and 29 (34.1%) were from Tongren Hospital. The mean age of the participants was 62.3 (SD 11.8) years, and 57% were male. The majority of the participants was married (85%) and retired (82%). The most common cause of renal failure was glomerulonephritis, followed by diabetic nephropathy. The average duration of dialysis was 4.9 (SD 3.7) years (Table 1). Twenty-nine participants completed the C-LoPAQ twice for test-retest reliability testing. The sociodemographic and clinical characteristics of the participants are displayed in Table 1.

**Table 1:** Demographic and clinical characteristics of the participants

Demographic characteristics	Validity test, ***n*** =85	Reliability test, ***n***=29
Age (Mean±SD)	62.3 ± 11.8	63.7 ± 10.7
Sex [(N (%)]		
Female	37 (43.5)	10 (34.5)
Male	48 (56.5)	19 (65.5)
Marital status [(N (%)]		
Married	72 (84.7)	26 (89.7)
Single	4 (4.7)	1 (3.4)
Widowed	7 (8.2)	1 (3.4)
Divorced	2 (2.4)	1 (3.4)
Employment [(N (%)]		
Full-time	2 (2.4)	0 (0)
Part-time	5 (5.9)	1 (3.4)
Retired	70 (82.4)	26 (89.7)
Others (farmers)	8 (9.4)	2 (6.9)
Educational level [(N (%)]		
Below senior middle school	39 (45.9)	14 (48.3)
Above senior middle school	46 (54.1)	15 (51.7)
Primary causes of renal failure [(N (%)]		
Chronic glomerular nephritis	43 (50.6)	10 (34.5)
Diabetes	9 (10.6)	5 (17.2)
Hypertension	5 (5.9)	4 (13.8)
Polycystic kidney	2 (2.4)	1 (3.4)
Lupus nephritis	3 (3.5)	1 (3.4)
Others and unknown	23 (27.1)	8 (27.6)
Dialysis duration, years (Mean±SD)	4.9 ± 3.7	8.9 ± 7.0
Laboratory parameters (Mean±SD)		
Serum creatinine, mg/dL	952.7 ± 280.8	966.6 ± 269.8
Hemoglobin, g/L	110.4 ± 17.8	116.6 ± 14.5
Serum albumin, g/L	38.9 ± 5.7	38.4 ± 4.6
Serum phosphorus, mmol/L	1.8 ± 0.6	1.8 ± 0.7
Serum iPTH, pg/dL	245.7 ± 182.3	282.4 ± 215.1
hs-CRP, mg/L	5.3 ± 8.9	6.5 ± 10.3

hs-CRP = high-sensitivity C-reactive protein; iPTH = intact parathyroid hormone

The overall mean step count using ActiGraph was 3807.8 (2723.0) steps/day, and 72.9% of the participants had an average step count of less than 5000 steps/day. In addition, 83.7% of the activity was light-intensity physical activity on average. The average energy expenditure was 986.1 (696.6) kcal/week. For the C-LoPAQ, the mean walk time was 28.4 (27.5) min/day. The total energy expended from walking and other activities was 1170.9 (820.1) kcal/week. Participants reported a mean sitting time of 4.7 h (Table 2).

**Table 2:** Objective and self-reported physical activity behavior (*n* = 85)

Variables	Mean ± SD
Total calories (kcal/week)	
C-LoPAQ	1170.9 ± 820.1
ActiGraph	986.1 ± 696.6
Physical activity	
C-LoPAQ, walking time (min/day)	28.4 ± 27.5
ActiGraph, step counts (step/day)	3807.8 ± 2723.0
Sedentary (min/week)	
C-LoPAQ, inactive time (min/week)	2393.6 ± 1440.9
ActiGraph, sedentary time (min/week)	5017.0 ± 2023.2

C-LoPAQ = Chinese version of the Low Physical Activity Questionnaire

### Validity estimate

For content validity, the expert panel commented that the use of “golfing” and “boating (motor)” as examples of “light activity” in the original English version of the LoPAQ was not appropriate, as Chinese MHD people do not usually engage in those exercises. Therefore, they were removed. Examples such as “light yard or gardening work” and “chair exercise” were replaced by “watering the plants” and “walking downstairs.” In the subscale of “moderate activity,” “aerobics class” and “swimming (the side stroke or breast stroke)” were replaced by “TaiChi” and “square dancing,” and “softball” and “downhill skiing” were deleted. In the subscale of “vigorous activity,” “playing tennis or racquetball” and “cross-country skiing” were removed and replaced with “slow rope skipping.” After the revisions, the panel rated the content validity of the C-LoPAQ using a 4-point Likert scale. The S-CVI was 0.91, and the CVR was 0.98.

With regard to criterion validity, Spearman coefficient analysis showed that there was moderate correlation between the C-LoPAQ and ActiGraph-measured physical activity for total calories (*rho* = 0.44, *p* < 0.01). The total walking time reported on C-LoPAQ was also correlated with step counts by ActiGraph (*rho* = 0.47, *p* < 0.01). There was a fair correlation between ActiGraph-measured sedentary time and C-LoPAQ-measured inactive time (*rho* = 0.22, *p* < 0.05) (Table 3).

**Table 3:** Spearman correlation analysis of C-LoPAQ and ActiGraph measurements (*n* = 85)

Physical activity measurements	Median	Inter-quartile range	Spearman Rank correlations
Total kcal per week			
C-LoPAQ	945	575–1664	0.44^*^
ActiGraph	837	525–1342	
Walking time/Step counts per week			
C-LoPAQ (Walking time, min)	165	60.0–262.5	0.47^*^
ActiGraph (Step counts)	23,497	13,475.5–37,685.5	
Sedentary time per week			
C-LoPAQ	2100	1575–3080	0.22^†^
ActiGraph	4404	3543–6797	

* Correlation is significant at the 0.01 level (2-tailed)

† Correlation is significant at the 0.05 level

C-LoPAQ = Chinese version of the Low Physical Activity Questionnaire

### Bland-Altman analysis

**Fig. 1:**
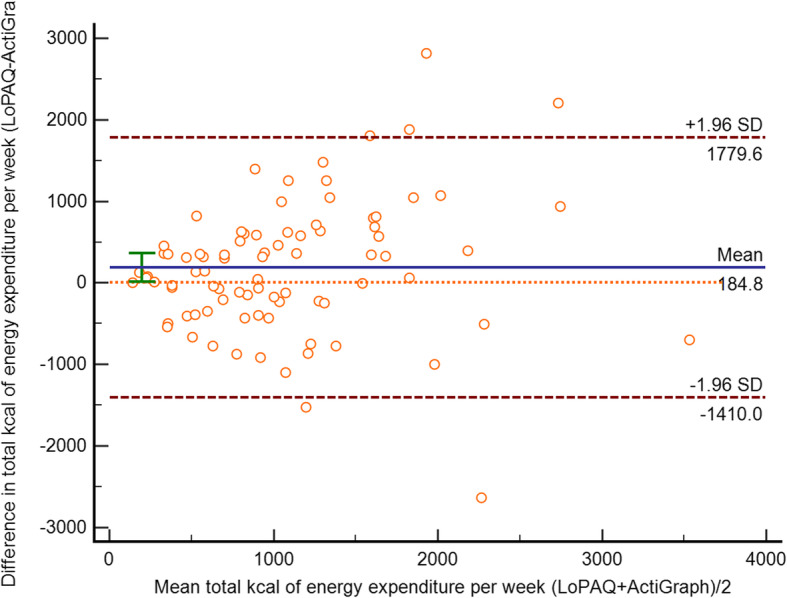
The Blant-Altman plot for total energy expenditure using ActiGraph and C-LoPAQ data

The results from the Bland-Altman plot show a mean difference of 184.8 kcal of energy expenditure per week between C-LoPAQ and ActiGraph data. In addition, MHD patients who were relatively active tended to over-report physical activity using the C-LoPAQ, and an error can be seen from over-reporting as the mean increases. The limits of agreement for total calories between the C-LoPAQ and ActiGraph had wide ranges, from − 1410.0 to 1779.6, with 6 outliers (7.1%) (shown in Fig. 1).

### Reliability estimate

The mean total energy expenditure of the C-LoPAQ was 1292.5 (SD 829.7) at the first test and 1732.7 (SD 1404.5) kcal/week at retest. For test-retest reliability, the ICCs ranged from 0.301 to 0.663 for the subscale scores. The details are shown in Table 4.

**Table 4:** Reliability of the C-LoPAQ subscales (*n* = 29)

C-LoPAQ scores	ICC
Total walking time, min/week	0.301
Energy expenditure in light activity, kcal/week	0.492
Energy expenditure in moderate activity, kcal/week	0.596
Energy expenditure in vigorous activity, kcal/week	0.663
Total energy expenditure, kcal/week	0.479
Sitting time, hours/day	0.432

C-LoPAQ = Chinese version of the Low Physical Activity Questionnaire; ICC = intraclass correlation coefficient

## Discussion

The aim of this study was to develop the C-LoPAQ and to determine its reliability and validity for measuring low levels of physical activity, especially walking, total kilocalories of physical activity, and time spent sitting compared with ActiGraph data from MHD patients. The main findings indicated that the walking times obtained by the C-LoPAQ moderately correlated with step counts measured by the ActiGraph. The total energy expenditure reported by the C-LoPAQ also correlated well with the kilocalories obtained from the ActiGraph. The C-LoPAQ-measured inactive time was associated with the ActiGraph-measured sedentary time. However, the C-LoPAQ had a fair test-retest reliability.

In the present study, 72.9% of MHD patients were sedentary (< 5000 steps/day) with a mean of 3807.8 (2723.0) steps/day. This finding is similar to a previous multicenter study in China, in which a mean daily step count of 3759.9 (2664.5) was documented among 320 MHD patients (mean age, 58.6 years) using pedometers [32]. In a national epidemiological study with 1163 dialysis patients (median age, 63 years) in France, Panaye et al. reported a median pedometer-measured physical activity of 3688 steps/day, and 64% of the participants were regarded as sedentary, walking < 5000 steps/day [33]. The levels of physical activity in this study may, therefore, be representative of MHD patients. Furthermore, we found that 83.7% of the activity was limited to light-intensity physical activity. Low physical activity is a potentially modifiable risk factor for disability and mortality [21]. These results confirm the importance of accurately capturing low levels of physical activity with simple, validated instruments, including the LoPAQ, among those undergoing dialysis.

It is important to assess the types of physical activities that are commonly engaged by the study population in a certain cultural context, as it is an indispensable part of content validity. Several examples of different intensities of physical activities were replaced with activities considered more culturally appropriate for Chinese dialysis patients’ lifestyles, or they were deleted altogether. For example, “aerobics class” was replaced by “TaiChi,” and “playing tennis or racquetball” was replaced by “slow rope skipping.” These replacements were achieved using activities with similar energy expenditure based on the Compendium of Physical Activities [34, 35]. The S-CVI was 0.91 and the CVR was 0.98, indicating excellent content validity.

The C-LoPAQ showed reasonable evidence of validity for walking time, as it was moderately correlated with ActiGraph-measured step counts, with a Spearman correlation (*rho* = 0.47) that was very close to that reported in a previous study [36]. Kittiskulnam et al. demonstrated that energy expenditure in walking according to the LoPAQ correlated with pedometer step counts, with a correlation coefficient of 0.53 [36]. The LoPAQ has the advantage of producing quantitative results that could be used to determine whether dialysis patients meet the recommended level of physical activity. The present study found an acceptable correlation (*rho* = 0.44) in measurements of total energy expenditure between the C-LoPAQ and accelerometer data. This result is relatively lower than that of a previous study, in which a correlation coefficient of 0.58 was found between the energy expenditures during walking measured by the LoPAQ and the Minnesota Leisure Time Activity Questionnaire [22]. In terms of absolute comparison of total energy expenditure between the C-LoPAQ and ActiGraph, a relatively large 95% limit of agreement from Bland-Altman analysis was found. Such an error is likely to be obvious in active respondents. This finding has been previously reported in questionnaires measuring physical activity. The IPAQ and Global Physical Activity Questionnaire were also found to have over-reporting issues as the mean levels of total physical activity or moderate-to-vigorous physical activity increased [37, 38]. It is important to point out that the accelerometer may not be able to accurately capture certain activities, such as biking, upper limb exercise, and gentle Taichi, which are popular in China.

Results from the current study demonstrated fair validity of the C-LoPAQ for sedentary behavior. Hours of sitting activity on the C-LoPAQ positively correlated with ActiGraph-measured sedentary time (*rho* = 0.22). However, the correlation did not reach the cut-off point (above 0.4) for what was generally considered acceptable validity. Such a low correlation may be due to different interpretations of sitting behavior among respondents. Some participants may not count lying down while not reading or watching television during waking time as sitting behavior. As suggested by Heesch et al. [39], it may be appropriate to add further details or examples that can enable respondents to fully comprehend the amount of time they spend sitting. Further, it should be noted that responses to sitting behavior may carry challenges associated with social desirability.

The test-retest reliability coefficients of the C-LoPAQ ranged from 0.30 to 0.66. Adequate test-retest reliability could be achieved if an individual is re-tested when they remain in a clinical steady state for the measured concepts. The lower test-retest reliability (ICC, 0.30) for walking time, compared to moderate and vigorous intensity activities, may be attributed to variability in walking behaviors in our study. In order to minimize the bias associated with learning or carryover effects from previous responses [40], a two-week interval for test-retest reliability was selected in this study. The retest did not cover the same period as the first C-LoPAQ test. Walking, both as a mode of transportation and fitness, was the major physical activity choice among the study participants. Previous studies documented an association between walking behavior and weather factors [41, 42]. This study was conducted in the winter. Considering the variables in weather (e.g., snow, rain, and windy) during the two-week time frame, the amount of walking behavior may be highly variable. Therefore, both true variability in walking activities and measurement error of C-LoPAQ over time may hamper its reliability.

A number of limitations should be addressed. First, even though the ActiGraph provides objective and valid measures of physical activity and sedentary behavior, it is not able to capture information on ambulatory activities, such as cycling, weightlifting, or water-based activities, such as swimming. This may overestimate sedentary behaviors. Second, a period of 2 weeks between the first and second tests may significantly affect the results of the reliability of the C-LoPAQ. Third, responsiveness or sensitivity to detect meaningful changes in the C-LoPAQ was not determined in the current study. Finally, as the size of the sample is relatively small, its generalizability might be an issue.

## Conclusions

The C-LoPAQ is easy to use and demonstrated an acceptable validity for measuring low levels of physical activity, especially walking, and total kilocalories of physical activity among dialysis patients in China. As a relatively low correlation was observed between sitting time in the C-LoPAQ and the ActiGraph-measured sedentary time, further clarification on the meaning of sitting time in the C-LoPAQ should be provided. Because the amount of physical activity may vary, re-testing of the C-LoPAQ should cover the same time period, consequently improving the accuracy of test-retest reliability. Further studies are needed to determine the responsiveness of the C-LoPAQ.

## Supplementary Information

**Additional file 1.**

## Data Availability

The datasets used and/or analyzed during the current study are available from the corresponding author upon reasonable request.

## Abbreviations

CKD: chronic kidney disease
ESRD: end-stage renal disease
MHD: maintenance hemodialysis
PD: peritoneal dialysis
PAR: Physical Activity Recall Questionnaire
IPAQ: International Physical Activity Questionnaire
LoPAQ: Low Physical Activity Questionnaire
C-LoPAQ: Chinese version of the LoPAQ
I-CVI: item-level content validity index
S-CVI: scale-level content validity index
CVR: content validity ratio
ICC: intraclass correlation coefficient
SD: standard deviation
