# Correction to: Validation of the Chinese version of the low physical activity questionnaire (LoPAQ) with ActiGraph accelerometer in hemodialysis patients

**DOI:** 10.1186/s12882-021-02245-w

**Published:** 2021-01-21

**Authors:** Rui Huang, Haifen Zhang, Yan Yang, Nina Fang, Qian Liu, Jun Ma, Min Wang, Ling Shi, Xingjuan Tao

**Affiliations:** 1 Shanghai Jiao Tong University School of Nursing, No.227, South Chongqing Rd, Shanghai, 200025 China; 2 Department of Biomedical Engineering, National University of Singapore, Singapore, Singapore; 3 Nursing Department, Renji Hospital, School of Medicine, Shanghai Jiao Tong University, Shanghai, China; 4 Department of Nephrology, Tong Ren Hospital, Shanghai Jiao Tong University School of Medicine, Shanghai, China

**Correction to: BMC Nephrol 22, 17 (2021)**

**https://doi.org/10.1186/s12882-021-02230-3**

Following publication of the original article [1], the authors informed us that the given name and family name of the authors were switched.

The author group has been updated above and the original article [1] has been corrected.
