# Supplementary material for: Validation of the Chinese version of the low physical activity questionnaire (LoPAQ) with ActiGraph accelerometer in hemodialysis patients
Source: BMC Nephrol. 2021 Jan 8;22:17. doi: 10.1186/s12882-021-02230-3 (PMC7791730; doi:10.1186/s12882-021-02230-3)
Supplement: Supplementary file 1 — Additional file 1. [file 12882_2021_2230_MOESM1_ESM.docx]

**Low Physical Activity Questionnaire (LoPAQ)**

1. Please check all of the statements that describe your walking in the last week

- I walked around the neighborhood

I walked ______ times during the week.

I walked for approximately ______ minutes per session.

- I walked for transportation (i.e. to the store, etc.)

I walked ______ times during the week.

I walked approximately ______ minutes per session.

- I walked for the purpose of improving my fitness or for pleasure (above and beyond daily necessities)

I walked ______ times during the week.

I walked approximately ______ minutes per session.

- I did not walk.

1. In the past 7 days, did you participate in other activities that would be described as "light" activities? These are activities that make your heart beat a bit faster than usual - you could talk and sing while doing them. Examples would be: light yard or gardening work, bowling, golfing, boating (motor), leisure bicycling, stretching, yoga, chair exercises or light housework such as folding laundry or washing dishes?

□ no □ yes

if yes, I participated in these light activities ______ times during the week for an average of ______ minutes per session.

1. In the past 7 days, did you participate in activities (other than walking) that would be described as "moderate" activities? These activities would make your heart beat faster than usual, and you could talk, but not sing while doing them. Examples of moderate activities would be: aerobics class, swimming (the side stroke or breast stroke), bicycling in the neighborhood, playing badminton or table tennis, softball, downhill skiing or moderate housework such as vacuuming or making beds.

□ no □ yes

if yes, I participated in these moderate activities ______ times during the week for an average of minutes per session.

1. In the past 7 days, did you participate in activities (other than walking) that would be described as "vigorous" activities? These would make your heart beat lots faster, make you breathe heavily, making your talking broken up by large breaths. Examples of vigorous activities would be: jogging or running, playing tennis or racquetball, playing soccer, basketball, cross country skiing, using stepping machines or other equipment at the gym.

□ no □ yes

if yes, I participated in these vigorous activities ______ times during the week for an average of ______ minutes per session.

1. In the past 7 days, did you do specific exercises for strengthening muscles?

□ no □ yes

if yes, I participated in these strengthening exercises ______ times during the week.

1. In the past 7 days, did you do exercises for stretching your muscles (flexibility exercises)?

□ no □ yes

if yes, I participated in these stretching or flexibility exercises ______ times during the week.

7. In terms of physical activity and walking, was this a typical week for you?

□ no □ yes

8. In the past 7 days, how much time did you spend sitting, watching television, reading or in front of a computer?

______ hours/day (average)

9. In the past 7 days, did you nap at home during the day?

□ no □ yes

if yes, I napped ______ times.

when you nap, how long do you nap? ______ minutes

10. In the past 7 days, how many hours were you in bed at night?

______ hours/night (average)

11. Do you work outside the home?

□ no □ yes

if yes, does your job require you to do walking?

□ no □ yes

if yes, your job require you to do physical exertion such as lifting?

□ no □ yes
